# Metabolic Syndrome, Insulin Resistance and Fatty Liver in Obese Iranian Children

**DOI:** 10.5812/ircmj.6656

**Published:** 2014-05-05

**Authors:** Forough Saki, Zohreh Karamizadeh

**Affiliations:** 1 Endocrinology and Metabolism Research Center, Shiraz University of Medical Sciences, Shiraz, IR Iran; 2 Division of Endocrinology, Department of Pediatrics, Shiraz University of Medical Sciences, Shiraz, IR Iran

**Keywords:** Insulin Resistance, Fatty liver, Obesity, Children, Iran

## Abstract

**Background:**

Obesity is a global epidemic and its morbidities such as metabolic syndrome, insulin resistance, and fatty liver leads to a spectrum of psycho-social and medical consequences.

**Objectives:**

The objective of this study was to investigate the prevalence of fatty liver in obese Iranian children and its' association with metabolic syndrome and insulin resistance.

**Patients and Methods:**

102 obese Iranian children, referred to pediatric clinics from March 2011 to March 2012, were enrolled in this cross-sectional study. All the patients were visited by a pediatric endocrinologist, a pediatric gastroenterologist and an expert radiologist in the evaluation of fatty liver grading.

**Results:**

The grade of fatty liver was higher in older children (P = 0.001). It was also more in taller and heavier children (P = 0.000). The more the BMI was, the more the fatty liver grade was (P = 0.002). Severity of fatty liver according to liver sonography in patient had a positive relationship with waist circumference, hip circumference, serum TG, serum FBS, serum fasting insulin, serum ALT, systolic blood pressure and HOMA index and had a negative correlation with the level of alkaline phosphatase. Severity of fatty liver also had a close relationship with the presence of acanthosis nigricans and HOMA index.

**Conclusions:**

Prevalence of fatty liver is high in our obese children. It was associated with criteria of metabolic syndrome and insulin resistance, so visceral fat may participate in the pathogenesis of the metabolic syndrome or merely serve as a marker of increased risk for the metabolic complications of obesity.

## 1. Background

Obesity has turned into a global epidemic in children and has a spectrum of psycho-social and medical consequences manifesting throughout the life span. Obesity and associated fat accumulation affects almost all organs of the body, but some systems like cardiovascular and endocrine are affected more than the others (1-5). Obese children have a high risk for developing hypertension, hyperlipidemia, atherosclerosis and diabetes mellitus type 2 (5). Since 1970s, Moran et al. identified chronic liver disease associated with obesity in adults as a distinct entity (6). Non-alcoholic fatty liver diseases (NAFLD) are one of these chronic disorders presented with lipid accumulation in the liver. This disease has a range of conditions involving the liver; the mildest type is simple fatty liver (hepatic steatosis), but there is a potentially serious type of non-alcoholic steatohepatitis (NASH), which is accompanied by liver-damaging inflammation and, sometimes, the formation of fibrosis. The more serious one is progressive fibrosis and cirrhosis (7). Investigators have used various methods to detect the prevalence rate of NAFLD in childhood. The presence, the degree or pattern of aminotransferase elevation are non-specific and cannot help us in etiological differentiation when used as a single method (2). Sonography of the liver has been found to be a good screening method in the evaluation of the degree of fat in the liver, having a sensitivity of 89% and specificity of 93% in detecting steatosis in the liver and a sensitivity of 77% and specifity of 89% in detecting increased fibrosis in the liver (8, 9). CT scan is more specific in detecting hepatic fibrosis, but it is costlier and not feasible at the moment for use in routine screening of suspected NAFLD (3, 9). Liver biopsy remains the gold standard for diagnosis of steatosis and various degrees of fibrosis, and for comparison of other various diagnostic modalities, but it is invasive and not used as a screening tool (9).

## 2. Objectives

Metabolic syndrome (MS) include a group of factors, which increase the risk of cardiovascular disease and is associated with insulin resistance (IR) and type 2 diabetes mellitus (10). The most commonly used definition was modified for metabolic syndrome in children from National Cholesterol Education program (NCEP) (11) in which children must have at least three of the following criteria:

1. Serum Triglyceride 110 mg/dL,
2. Serum HDL-C 40 mg/dL,
3. Fasting blood sugar 100 mg/dL,
4. Waist circumference 90th percentile for age and gender, and
5. blood pressure 90th percentile for age and sex (11).

Importance of obesity as a global epidemic and its morbidities, most importantly metabolic syndrome, insulin resistance, fatty liver, lack of data about the prevalence of fatty liver and its predictors in obese Iranian children prompted us to do this study.

## 3. Patients and Methods

### 3.1. Patients

From 141 obese Iranian children, who referred to Shiraz University Medical Pediatric Clinics during March 2011 to March 2012, 102 were enrolled in this cross-sectional study according to the inclusion and exclusion criteria. Shiraz is the capital city of Fars province in south of Iran.

The inclusion criteria were:

1. BMI higher than 95 percentile for their age, and sex,
2. Age between 5-17 years,
3. confirmation to participate in our study after discussing the aim and method of the study in an oral presentation and signing the informed consent.

The exclusion criteria were:

1. liver disease except NAFLD (such as viral hepatitis, autoimmune hepatitis, Wilson disease, α1-antitrypsin deficiency, hemochromatosis),
2. renal failure,
3. trauma,
4. acute illness,
5. using any medication causing changes in serologic tests,
6. diabetes mellitus (FBS 126 or random blood sugar more than 200).

All the patients were visited by a pediatric endocrinologist, who measured Children’s height and weight. He measured height with a wall-mounted meter with the child standing without shoes, and rounded to the nearest 0.5 cm. Also, he measured weight was with one standard balance (Seca, Germany) then rounded to the nearest 0.1 kg. Body mass index (BMI) was calculated via this formula: BMI (kg/m^2^) = weight (kg)/ [Height (m)]^ 2^. A pediatric gastroenterologist and an expert radiologist evaluate the fatty liver grading. Data on height, weight, waist circumference (WC), hip circumference (HC) blood pressure, puberty tanner staging and clinical evidence of insulin resistance (e.g. acanthosis nigricans) of the patients were gathered by a pediatric endocrinologist. NAFLD was diagnosed in children according to liver enzymes and liver sonography by a pediatric gastroenterologist. Blood sample was obtained from each patient at the time of liver sonography, and they were all checked in a single expert laboratory to check serum triglyceride (TG), cholesterol (Chol), high density lipoprotein (HDL), low density lipoprotein (LDL), fasting glucose, fasting insulin level, HbA1c, uric acid, ALT, and AST. Neither the physicians nor the radiologist knew the patients’ laboratory data. The degree of insulin resistance was determined by the hemostatic model assessment (HOMA) using the formula (12): fasting insulin (µu/mL) x fasting glucose (mmol/L)/22.5.

According to previous studies prevalence of fatty liver in obese Iranian children was 54.4% (12). Statistical specialist calculated that with expected power = 80% we can do our study on 98 obese children.

### 3.2. Liver Sonography for Fatty Liver Grading

The degree of liver steatosis was graded from zero (no steatosis) to 3 according to savergmutta, (13) based on ultrasonographic examination using General Electric LOQIQ 500, convex 3.5 MHZ. In grade 1 (mild), echogenecity is slightly increased, with normal visualization of the diaphragm and the intrahepatic vessel borders. In grade 2 (moderate), echogenecity is moderately increased, with slightly impaired visualization of the diaphragm or intrahepatic vessels. In grade 3 (severe), echogenecity is markedly increased, with poor or no visualization of the diaphragm, the intrahepatic vessels and posterior portion of the right lobe.

### 3.3. Ethics

All patients and their parents signed the informed consent forms after an oral-presentation about the aim and method of the study. The study was approved by medical ethics committee of Shiraz University of Medical Sciences with number 4086 in 2011.6.8.

### 3.4. Data Analysis

Statistics analysis was performed for all variables using SPSS, version 15. All the parameters were expressed as mean value ± standard deviation (S.D.). The normal assumption of data was checked by one sample Kolmogorov-Sinirnov test. If it is normal, comparison was done using multivariate test and analysis of variance (ANOVA) test, and if it is not normal for example for ALT and alkaline phosphatase Kuskal-Wallis test were used to use to compare the grade of fatty liver and other nonparametric data. The P values less than 0.05 was considered as statistically significant.

## 4. Results

This study was conducted on 102 obese children aged 5-17 years, with a mean age of 10.57 ± 3.04 years. 66 (64.7%) children were female. Six patient’s blood samples failed for the tests. All these 6 patients were recalled for resampling. Four of them were rechecked, but two of them did not refer for resampling. We ignored them after consulting with our statistical specialist because it is so small (< 2%). Their BMI was 30.29 ± 8.2. All other results are summarized in Table 1. Forty six patients (45.1%) did not have fatty liver according to sonography. Forty four of them (43.1%) were grade I and 12 patients had grade II sonographic fatty liver. None of them had grade III fatty liver in ultrasonographic evaluation. All data in each grade of fatty liver in patients and also the P value of the association of these data with the grade of fatty liver are summarized in Table 1. The grade of fatty liver was higher in older children (P = 0.001). It was also more in taller (P < 0.001) and heavier children (P < 0.001). The more the BMI was, the more the fatty liver grade was (P = 0.002). Severity of fatty liver according to ultrasonography evaluation of the liver in patients had a positive relationship with waist circumference (P < 0.001), hip circumference (P < 0.001), serum TG (P = 0.001), serum FBS (P = 0.002), serum fasting insulin (P = 0.001), serum ALT (P = 0.017), systolic blood pressure (P = 0.048), and HOMA index (P < 0.001) and it had a negative correlation with the level of alkaline phosphatase (Alk) (P = 0.041). There was no correlation between the grade of fatty liver and WC/HC ratio, serum cholesterol, serum HDL, LDL, HbA1C, uric acid, AST and Diastolic blood pressure (Table 1). Among 46 patients who had not fatty liver, 12 patients had criteria of metabolic syndrome (26%); from 44 patients with grade 1 fatty liver 30 had criteria of metabolic syndrome (68%), and all patients with grade II fatty liver had metabolic syndrome (100%). There is a close relationship between the grade of fatty liver and prevalence of metabolic syndrome (P < 0.001). Also, waist circumference, hypertriglyceridemia, hyperglycemia and elevated systolic blood pressure were the most prevalent criteria in our patients in diagnosis of metabolic syndrome, respectively. Low HDL was seen just in 8 patients with grade II fatty liver, and 3 patients with grade I fatty liver. All data about metabolic syndrome are summarized in Table 2. Severity of fatty liver also had a close relationship with clinical signs of acanthosis nigricans and HOMA index, the signs of insulin resistance.

**Table 1.** Data Separated by the Grade of Fatty Liver in Sonography and P Value of Correlation of Fatty Liver Severity and the Data^a,b,c^

Data	Fatty Liver Grade			
	Grade 0	Grade 1	Grade 2	P value
**Age, y**	8.96 ± 2.48	11.31 ± 2.7	14 ± 2.36	0.001
**Height, cm**	134 ± 12.11	144.95 ± 16/94	160.83 ± 9.2	< 0.001
**Height Z score**	1.56 ± 0.67	1.67 ± 0.64	1.73 ± 0.78	0.045
**Weight, kg**	47.86 ± 10.99	67.04 ± 26.86	99.16 ± 25.60	< 0.001
**Weight Z score**	2.34 ± 0.46	2.65 ± 0.58	2.81 ± 0.56	0.009
**BMI** **, kg/m** ^**2**^	26.83 ± 2.27	31.55 ± 10.61	38.91 ± 5.21	0.002
**Waist circumference, cm**	78.69 ± 8.17	90.54 ± 14.60	109.5 ± 18.37	< 0.001
**Hip circumference, cm**	83.39 ± 8.17	94.40 ± 12.7	116.1 ± 19.46	< 0.001
**WC/HC**	0.941 ± 0.02	0.955 ± 0.03	0.95 ± 0.01	0.322
**TG, mg/dL**	105.6 ± 41.6	97.9 ± 42.5	241 ± 216	0.001
**Chol, mg/dL**	156.6 ± 25.72	153.7 ± 30	165.5 ± 39.7	0.157
**HDL, mg/dL**	45.7 ± 9.8	46.45 ± 8.6	38.6 ± 15.17	0.263
**LDL, mg/dL**	87.13 ± 17.5	85.27 ± 21.8	90.16 ± 25.1	0.317
**FBS, mg/dL**	89.08 ± 9.4	95.5 ± 7.07	103.3 ± 12.8	0.002
**Fasting Insulin, µIu/mL**	13.88 ± 7.8	23.75 ± 15.9	37.91 ± 18.11	0.001
**HbA1C, %**	5.74 ± 0.52	5.8 ± 0.54	6.07 ± 0.808	0.057
**Uric acid, mg/d**	4.35 ± 1.1	5.15 ± 1.45	5.9 ± 0.5	0.069
**ALT, Iu/L**	24.6 ± 9.39	37.36 ± 56.69	55.83 ± 68.8	0.017
**Alkaline phosphatase**	588.4 ± 230	504.3 ± 120.6	379.16	0.04
**Systolic BP** ^**c**^ **, mmHg**	109.5 ± 13.3	115 ± 15	123 ± 8.16	0.048
**Diastolic Bp, mmHg**	71.73 ± 7.77	70.68 ± 4.95	70 ± 0.12	0.218
**HOMA index**	1.27 ± 0.74	2.31 ± 1.55	4 ± 2.3	< 0.001

^a^ Data were summarized as mean ± SD

^b^ There is no grade 3 fatty liver in our patients

^c^ Abbreviation: ALT, alanine transaminase; BMI, body mass index; Bp, blood pressure; Chol, Cholesterol; FBS, fasting blood sugar; HbA1C, hemoglobin A1C; HC, hip circumference; HDL, high density lipoprotein; LDL, low density lipoprotein; TG, triglyceride; WC, waist circumference

**Table 2.** Criteria of Metabolic Syndrome in Each Grade of Fatty Liver^a^

Criteria	Grade of Fatty Liver			
	Grade 0	Grade 1	Grade 2	Total
**Waist circumference 90th percentile**	13 (28.2)	28 (63.6)	12 (100)	53 (51.9)
**Serum triglyceride 110 mg/dL**	13 (28.2)	23 (52.2)	12 (100)	48 (47)
**HDL-C** ^**b**^ **40 mg/d**	1 (2.1)	3 (6.8)	8 (66.6)	12 (11.7)
**Fasting glucose 100 mg/dL**	12 (26)	20 (45)	9 (75)	41 (40.2)
**Case of metabolic syndrome**	12 (26)	30 (68)	12 (100)	54 (52.9)
**Total**	46 (45.1)	44 (43.1)	12 (11.8)	102 (100)

^a^ Data presentation are No. (%).

^b^ Abbreviation: HDL-C, high density lipoprotein.

## 5. Discussion

Among our 102 obese children, prevalence of sonographic fatty liver was 54.9%. It was more prevalent in older, taller, heavier and the patients having more BMI. Possibility and severity of fatty liver in sonography was more in patients that had more waist circumference, more hip circumference, more serum TG, more serum FBS, more serum fasting insulin, more serum Alt, more systolic blood pressure, more HOMA index, and less serum alkaline phosphatase. There is a close correlation between severity of fatty liver and signs of insulin resistance (HOMA index, fasting blood sugar, fasting insulin level and clinical evidence of acanthosis nigricans). Also, prevalence of metabolic syndrome in all our obese children was 52.9%. 100% of grade II fatty liver, 68% of grade I fatty liver and 26% of patients without evidence of fatty liver in sonography had criteria of metabolic syndrome. It seems that metabolic syndrome has a close association with the presence of fatty liver. Among the criteria of metabolic syndrome in children with fatty liver, increased waist circumference, hypertriglyceridemia, high fasting blood sugar and high systolic blood pressure were more prevalent, respectively. It was observed that fatty liver in obesity; insulin resistance and metabolic syndrome were parts of a chain with a tight connection, having a cause and effect relationship. It is unclear whether visceral (omental and mesenteric) or subcutaneous deposits of abdominal fat are more closely related to insulin resistance, because data from different studies are contradictory. In addition, visceral fat mass often correlates with subcutaneous fat mass, so it is difficult to separate the contribution of each depot to insulin resistance. The ectopic distribution of triglycerides in nonadipose tissue is also closely correlated with the metabolic complications of obesity. Data from a series of studies showed that insulin resistance to glucose metabolism in skeletal muscle is correlated with the intramyocellular concentration of triglyceride. In addition, excessive intrahepatic triglyceride content is associated with serious cardiometabolic abnormalities, including T2DM, dyslipidemia (high plasma triglyceride, low plasma HDL-C, or both), hypertension, the dysmetabolic syndrome, and coronary heart disease. It is not known whether triglycerides interfere with insulin action or whether triglycerides serve as surrogate markers for some other fatty acid–derived entity (from plasma or intracellular sources) that impairs insulin signaling (6, 10-19).

Among the previous studies, data on the prevalence of NAFLD in obese children were scanty. These studies were performed mainly in tertiary medical centers, and it has been reported to a range from 20-77% (2, 10, 15). In an Italian multi-center study, abnormal liver enzyme tests were seen in 10-15% of obese children (2, 16). Also in that country, 42% of obese children had steatosis by sonography and presence of fatty liver was correlated well with BMI (2, 17).

Evidence of hepatic steatosis in obese children in China was 77% (14) and these children had greater insulin resistance (2, 14). In a large multiethnic origin in Canadian obese children, the prevalence of fatty liver was 71% and cirrhosis was seen in one. About 30% of them had insulin resistance and acanthosis nigricans (18). Their data were close to our data, so prevalence of fatty liver in obese children in multiple countries was the same, being about 45-77%.

In some relevant studies, similar to our results, it seems that the sequel of hepatic steatosis becomes more apparent and more prevalent in older obese children (19, 20). Schwimmer et al. in 2003 showed that obesity was seen in 88% of children with NAFLD. Also, he showed that fasting hyperinsulinemia was present in 75% of children with NAFLD and insulin resistance (assessed by HOMA index) was present in 95% of the subjects (20). In this study, he concluded that children with NAFLD should be screened for insulin resistance which is nearly universal, and correlates well with liver histology. In a study in Egypt, 54.2% of the obese children had criteria of metabolic syndrome, and the prevalence of NAFLD in their obese children was 45%. They concluded that there is a close association between obesity, metabolic syndrome and NAFLD (10). Ascaso et al. showed that abdominal obesity appears to be a good indicator of risk for insulin resistance and the metabolic syndrome in adults particularly in non-obese ones (21).

Furthermore, some studies revealed the importance of obesity in Iranian children and showed that the high prevalence of hypertension in overweight and obese children emphasizes the need for prevention and control of childhood obesity and hypertension in early stages (22-24).

According to our study as well as the previous ones, it seems that non-alcoholic fatty liver associated with obesity is now well established in children as a major cause of chronic liver disease, and it is the same in Asia and Europe, among different countries. Also, there is a close relationship between insulin resistance and metabolic syndrome, which increases the risk of cardiovascular disease. This condition is potentially reversible and preventable. Large multicenter programs are required to control and prevent obesity in children and these important morbidities. There is an urgent need to do large population based studies in children from ethnic and regional areas, to document the epidemic of obesity. Trends of the changes will help health managers and policymakers to do proper interventions.

Our strong point is that we investigate 3 main prevalent metabolic disorders in Iranian children, obesity, and fatty liver and metabolic syndrome, also we evaluate their association. However, a weak point of our study is that we could not evaluate the molecular base of these important diseases, so we could not mention an exact causal relationship between them. More study need to evaluate the cause effect relationship between these important metabolic problems.

It was shown that the prevalence of fatty liver is high in our obese children. It was associated with metabolic syndrome and insulin resistance, so visceral fat actually participates in the pathogenesis of the metabolic syndrome or merely serves as a marker of increased risk for the metabolic complications of obesity. Also, we showed that the severity of fatty liver was higher in those obese children that had more height, more weight, more BMI, more waist and Hip circumference, more serum triglyceride, more serum FBS, more serum fasting insulin, more systolic blood pressure, and less serum alkaline phosphatase level. Furthermore, as to the criteria of metabolic syndrome, hypertriglyceridemia and high waist circumference were more prevalent in our obese children with fatty liver. These conditions are potentially reversible and preventable. More studies should be done to know whether triglycerides interfere with insulin action or whether triglycerides serve as surrogate markers for some other fatty acid–derived entity that impairs insulin signaling.
